# The National Dental Association

**Published:** 1898-07

**Authors:** 


### THE NATIONAL DENTAL ASSOCIATION.

THIS body adjourned at Old Point Comfort to meet at Omaha; but it is learned from the chairman of the Executive Committee that it is thought this selection was not a wise one, and efforts are, at this writing, about to be made to secure the consent of the members to a change. When it is considered that eighty-six organizations are advertised to meet at Omaha during the continuance of the exhibition, it does seem as though some action was necessary.

The question, Where shall the National go? will assume first importance to the members in the next few weeks. Probably before this reaches our readers it will have been decided. The choice is limited, and, it is presumed, will lie between Chicago and Denver. While the former is convenient, and possesses many advantages, it certainly will not meet the views of many, and especially those resident on or near the Pacific coast. One of the objects in locating the meeting this year West was to accommodate these, and it is due to them that it should be held as far west as possible. Denver meets this want, and it is certainly to be desired that this place be selected for the meeting of the national organization as well as all other contributing bodies.

